# The Motile Breast Cancer Phenotype Roles of Proteoglycans/Glycosaminoglycans

**DOI:** 10.1155/2014/124321

**Published:** 2014-07-22

**Authors:** Dragana Nikitovic, Katerina Kouvidi, Kallirroi Voudouri, Aikaterini Berdiaki, Evgenia Karousou, Alberto Passi, George N. Tzanakakis

**Affiliations:** ^1^Laboratory of Anatomy-Histology-Embryology, Medical School, University of Crete, 71003 Heraklion, Greece; ^2^Dipartimento di Scienze Chirurgiche e Morfologiche, Università degli Studi dell'Insubria, Via J.H. Dunant 5, 21100 Varese, Italy

## Abstract

The consecutive stages of cancer growth and dissemination are obligatorily perpetrated through specific interactions of the tumor cells with their microenvironment. Importantly, cell-associated and tumor microenvironment glycosaminoglycans (GAGs)/proteoglycan (PG) content and distribution are markedly altered during tumor pathogenesis and progression. GAGs and PGs perform multiple functions in specific stages of the metastatic cascade due to their defined structure and ability to interact with both ligands and receptors regulating cancer pathogenesis. Thus, GAGs/PGs may modulate downstream signaling of key cellular mediators including insulin growth factor receptor (IGFR), epidermal growth factor receptor (EGFR), estrogen receptors (ERs), or Wnt members. In the present review we will focus on breast cancer motility in correlation with their GAG/PG content and critically discuss mechanisms involved. Furthermore, new approaches involving GAGs/PGs as potential prognostic/diagnostic markers or as therapeutic agents for cancer-related pathologies are being proposed.

## 1. Introduction

*Cancer Microenvironment*. It is now increasingly recognized that the microenvironment plays a critical role in the progression of tumors. The consecutive steps of tumor growth, local invasion, intravasation, extravasation, and invasion of anatomically distant sites are obligatorily perpetrated through specific interactions of the tumor cells with their microenvironment. Free glycosaminoglycans (GAGs) and proteoglycan- (PG-) containing GAGs, key effectors of cell surface, pericellular and extracellular microenvironments, perform multiple functions in cancer by virtue of their coded structure and their ability to interact with both ligands and receptors that regulate cancer growth [1–4]. Specifically, these extracellular matrix (ECM) components critically modulate the tumor cell “motile phenotype” affecting their adhesive/migratory abilities which are directly correlated to the metastatic cascade [5, 6].

Glycosaminoglycans (GAGs) comprise a class of linear, negatively charged polysaccharides composed of repeating disaccharide units of acetylated hexosamines (N-acetyl-galactosamine in the case of chondroitin sulphate and dermatan sulfate or N-acetyl-glucosamine in the case of heparin sulphate and heparin) and mainly of uronic acids (d-glucuronic acid or l-iduronic acid) being sulfated at various positions. The exception constitutes keratan sulphate whose uronic acid is substituted by galactose. Based on the epimeric form of uronic acid and the type of hexosamine in their repeating disaccharide units, GAGs are classified into four major types; hyaluronan (HA), chondroitin sulfate (CS) and dermatan sulfate (DS), heparin and heparan sulphate (HS), and keratan sulfate (KS). HA is synthesized in the absence of a protein core at the inner face of the plasma membrane and consequently found in the form of free chains whereas other GAG types are covalently bound into protein cores to form proteoglycans (PGs). With the exception of HA, all GAG types are variably sulfated which contributes to the intricate complexity of their structures. Free GAGs chains are secreted to the extracellular space and distributed both in the pericellular matrix and extracellular matrix proper. GAGs bound into PGs are located to the extracellular matrix, basal membrane, and cell surface [7]. Cell type and tissue specific alterations in fine GAG structure, which are strictly predetermined [8–10], allow these molecules to modulate with high specificity different cellular processes [7]. Cell-associated and tumor microenvironment GAG content and distribution is markedly altered during tumor pathogenesis and progression [11, 12].

PGs, molecules which consist of a protein core that is covalently modified with GAG chains, are distributed both to the ECM “proper” associated with the cell membrane as well as located to intracellular compartment. These main PG groups are further classified into families according to their gene homology, core protein properties, size, and modular composition. Thus, secreted to the ECM PGs include large aggregating PGs, named hyalectans, small leucine-rich PGs (SLRPs), and basement membrane PGs. Cell-surface-associated PGs are distributed into two main families (syndecans and glypicans), whereas serglycin is the only intracellular PG characterized to date [13, 14]. The wide molecular diversity of PGs is derived from the multitude of possible combinations of protein cores and GAG chains. Thus, PGs are also classified, regarding their GAG content, into heparan sulfate PGs (HSPG), chondroitin/dermatan sulfate PGs, (CS/DSPGs), and keratan sulphate PGs. The specific structural characteristics of both the protein cores and GAG types provide the structural basis for the plethora of their biological functions which include acting as structural components in tissue organization or dynamic regulators of cellular behaviour [3].

## 2. Is the Expression of PGs/GAGs in Breast Cancer Correlated to Disease Progression?

Importantly, ECM components, including PGs and GAGs, are involved in the molecular events that are associated with tumor progression. It is well established that during malignant transformation, significant changes can be observed in the structural and mechanical properties of respective ECM components. Indeed, the alteration of cell shape and changes in the interactions with the ECM are considered as important hallmarks of cancer cells [15, 16]. Changes in the composition and organization of ECM regulate cancer progression by promoting cellular transformation and metastasis. Moreover, altered expression of ECM molecules also deregulates the behavior of stromal cells and promotes tumor-associated angiogenesis and inflammation, leading to the generation of a tumorigenic microenvironment [17–19].

HSPGs have been closely correlated to breast cancer tumorigenesis. Major HSPGs members are the transmembrane proteins syndecans (SDCs), with the SDC family consisting of four members: SDC1, SDC2, SDC3, and SDC4 [20]. A complex pattern describing SDCs' expression in tumor and stroma compartments during the progression of malignancy is emerging. Most reports have focused on the involvement of SDC1, an epithelial marker, during the progression of this insidious disease. Thus, increased expression of SDC1 was demonstrated in the stroma of invasive breast cancer [21–23]. Moreover, the expression of SDC1 in both epithelium and stroma may be a predictor of unfavorable prognosis in breast cancer, whereas loss of epithelial SDC1 was associated with a more favorable outcome [21]. Importantly, SDC1 has also been linked with the promotion of proliferation of human breast cancer cells* in vitro* [23]. The distribution of SDC1 to cell membrane has predominantly been described in breast cancer; however, shed SDC1 in other tumor types has been directly associated with increased invasion and cancer progression [24, 25]. Indeed in breast cancer, SDC1 is suggested to be a poor prognostic factor for breast cancer since its upregulation at both the mRNA and protein levels has been associated with higher histological tumor grade, as well as increased mitotic index and tumor size [26]. The expression of other SDC family members in breast cancer tissues has also been studied. Thus, in estrogen receptor-negative and highly proliferative breast carcinoma subtypes, SDC1 and SDC4 were found to be overexpressed [27]. Similarly, the overexpression of these two PGs has been demonstrated in a highly invasive breast cancer cell line (MDA-MB-231) [28]. However, another report suggests that SDC4 expression is downregulated in malignant breast tissue [29]. The data on SDC1' roles seem to be more uniform as high expression of SDC1 has been linked with increased tumor aggressiveness and poorer prognosis in breast carcinomas [30]. Functionally, this correlates well with the proposed role of SDC1 as a coreceptor which activates mitogenic growth factor signaling which in turn modulates tumor angiogenesis, cell adhesion, and motility [31]. Moreover, a study conducted in postmenopausal women with breast cancer or dense-mammographic breast tissue demonstrated that the distribution of SDC1 changes from the epithelium to the stroma [32, 33]. Interestingly, SDC1 expressing breast carcinomas show decreased response to chemotherapy [34], whereas it has also been indicated that the loss of SDC1 expression may be a potential predictive factor for response to preoperative systemic therapy [35]. These data define SDC1 as a potentially significant therapy target.

The glypicans (GPCs) are HSPGs anchored through the glycosylphosphatidylinositol (GPI) link to the outer layer of cell membranes. GPCs have been shown to regulate the binding properties of bone morphogenetic protein (BMP) and fibroblast growth factor (FGF) [36]. Most of the studies concerning the roles of GPCs in breast cancer progression focus on the role of the GPC3 member. Intriguingly, the GPC3 gene silencing has been identified in human breast cancer cells, through a mechanism which involves the hypermethylation of the GPC3 promoter. Thus, GPC3 seems to be a negative regulator of breast cancer cell proliferation, since it was shown that its ectopic expression inhibited the growth rates of 8 in a panel of 10 breast cancer cell lines [37]. Furthermore, it has been established that GPC3 guides MCF-7 breast cancer cells to apoptosis through a mechanism that involves the anchorage of the GPC3 core protein to the cell membrane [38]. The role of the other members of the GPC family in breast cancer pathogenesis has not been widely investigated. The up to now obtained data suggest that the expression of GPC3 and GPC4 was negligibly increased in tumor as compared to normal tissues, whereas the expression of GPC5 and GPC6 was below the level of detection in both normal and cancerous breast tissues. On the contrary, in the same study GPC1 was found to be strongly expressed in human breast cancers with a low expression in normal breast tissues [39].

The family of PGs secreted to the ECM and known as hyalectans is comprised of versican, aggregan, neuroscan, and brevican [12]. Versican seems to have a prominent role in breast cancer progression due to its ability to interact with molecules determined to be regulators of key cellular processes [40]. Importantly, extracellular versican has been found to be elevated in a variety of human tumors including breast carcinoma [41–43]. The distribution of versican in tissue samples is mostly allocated to breast cancer margins. Indeed, the high expression of versican has been described in the interstitium at the invasive margins of breast carcinoma. Versican is suggested to be a prognostic marker as it has been found to be predictive of cancer relapse, negatively affecting overall survival rates of breast cancer patients [44]. On the other hand, the increased expression of versican within peritumoral stromal matrix was predictive of relapse-free disease prognosis, in women with node-negative breast cancer. These authors therefore propose that versican may be a predictor for risk and rate of relapse, independent of tumor size in patients with node negative disease [45]. Recently, various histotypes of breast* in situ* carcinomas have been examined in order to assess the immunohistochemical expression of versican in the stroma and correlate these findings to disease progression. This study provided evidence that versican is strongly expressed in the perilesional stroma of a subclass of ductal* in situ* carcinomas and that the extension of versican immunostaining is statistically related to the high grade. On the other hand, the expression of versican in the cases of classic lobular* in situ* carcinomas was confined to the anatomical structures that usually contain this PG in adult breast tissues [46]. Thus, Canavese et al. suggest that various histotypes of breast* in situ* carcinomas could follow different pathways of epithelial stromal interactions. Structure-function studies focusing on versican suggest that its G3 domain is closely correlated to breast cancer progression. Thus expression of versican G3 domain both increases breast cancer cell proliferation* in vitro *and* in vivo* and also enhances tumor cell migration* in vitro* and systemic metastasis* in vivo* [47, 48]. The exogenous expression of a versican G3 construct in breast cancer cell lines enhanced their resistance to anthracycline-dependent apoptosis when cultured in serum free medium by upregulating pERK and GSK-3b (S9P) [49]. On the other hand, versican G3 promoted cell apoptosis induced by C2-ceramide or Docetaxel by enhancing expression of pSAPK/JNK and decreasing expression of GSK-3*β* (S9P). Inhibition of endogenous versican expression by siRNA or reduction of versican G3's expression by linking G3 with 3′UTR prevented G3 modulated cell apoptosis. Thus, the G3 domain appears to have a dual role in modulating breast cancer cell resistance to chemotherapeutic agents [49]. The importance of versican in breast cancer pathogenesis is well illustrated in a recent study by Kischel et al. These authors demonstrate that all known versican isoforms as well as new alternatively spliced versican isoform, named V4, were significantly overexpressed in the malignant lesions [50].

The small leucine-rich proteoglycans (SLRPs) are characterized by a relatively small protein core with leucine rich-repeat (LRR) motifs into which GAG chains are covalently bound [13, 51, 52]. These secreted proteins have the ability to interact with collagen, modifying the deposition and organization of collagen fibers in the extracellular matrix. A study on SLRP expression in breast tumors showed that lumican and decorin are the most frequently expressed SLRPs, whereas biglycan and fibromodulin are rarely detected [53]. Decorin is physiologically secreted by stromal fibroblasts of normal breast tissue [54]. Indeed, the expression of decorin, which is abundant in the stroma, can be used as an indicator of tumor progression [55]. Specifically, low expression of decorin has been correlated to large tumor size, a shorter time to progression, and poorer survival [55]. In a study by Reed et al., it has been shown that the primary tumor growth was strongly diminished after treatment with decorin protein core. In the same study, the utilization of an adenoviral vector containing the decorin transgene caused the elimination of metastases [56]. Decorin has also been shown to decrease tumor growth in experiments conducted in a rat model [56]. Moreover, it has been indicated that decorin inactivates the oncogenic ErbB2 protein [57]. Another important member of the SLRP family, lumican, is specifically expressed in breast cancer tissues, but not in normal breast tissues. Furthermore, it has been proposed that lumican is differentially expressed during breast tumor progression [58]. The overexpression of lumican in breast cancer tissues is associated with a high tumor grade, a low estrogen receptor (ER) expression level, and young age of patients [58].

Hyaluronan (HA) is an anionic, nonsulfated GAG which differs from the other members of the GAG family as it neither contains sulfate groups nor is it covalently linked into a core protein [59]. This GAG is synthesized by three types of integral membrane proteins denominated hyaluronan synthases: HAS1, HAS2, and HAS3. The degradation of HA within tissues, on the other hand, is performed by enzymes known as hyaluronidases (HYAL). A significant number of studies demonstrate that HA deposition is elevated in various types of cancer tissues including breast cancer [60]. Specifically, immunochemistry revealed elevated amounts of HA in the stroma of human breast cancer, correlating with tumor invasion, metastasis, and adverse clinical outcome [61, 62]. The magnitude of the HA accumulation in the tumor stroma (breast, ovarian, and prostate cancers) strongly correlates with an unfavorable prognosis of the patient, that is, advancement of the malignancy [59]. HYAL1 and HYAL2 are found to be overexpressed in breast cancer tumors, downregulating the expression of HA [63].

Taking into consideration all the above, it can be concluded that PGs/GAGs, which are abundantly present in the stromal compartment of breast cancer cells, play a major role in several biological processes of carcinogenesis. The overexpression of many of these molecules has been associated with the malignant phenotype and with poor prognosis. The* de facto* contribution of these molecules to tumor cells' malignant properties defines them as relevant therapeutic agents.

## 3. The “Motile” Phenotype

Tumors of solid organs (carcinomas, sarcomas, and central nervous system tumors) kill patients mainly by dissemination from the primary site as once the cells migrate beyond the primary site into adjacent or distant tissue, they are difficult to extirpate. This dissemination may take two forms: (i) localized invasion throughout the tissue and into the adnexa or (ii) metastatic dissemination [64]. An obligatory component of the dissemination process is the obtaining of a “motile phenotype.” In order for the tumor cells to efficiently migrate, specific cytoskeleton modifications must be executed. First, actin cytoskeleton organization has a well-established role in cell migration and is regulated by a plethora of extensively studied molecular mediators. Specifically, Rho GTPases, cAMP/PKA, and integrins were found to have a central role in modulating the actin cytoskeleton alterations during migration and have been shown to be closely regulated during epithelial to mesenchymal transition (EMT) processes [65, 66]. Integrins are heterodimeric cell-surface molecules that on one side link the actin cytoskeleton to the cell membrane and on the other side mediate cell-matrix interactions [67]. In addition to their structural functions, integrins mediate signaling from the extracellular space into the cell through integrin-associated signalling and adaptor molecules such as FAK (focal adhesion kinase) [68] or ILK (integrin-linked kinase) [69]. Intermediate filaments (IFs) play a central role in maintaining cell structure, stiffness, and integrity. The IF network of epithelial cells comprises cytokeratins, while the mesenchymal IF network is primarily constituted of vimentin. During EMTs, many cytokeratins are downregulated and vimentin is upregulated [70]. Overexpression of vimentin IFs in the breast carcinoma model leads to augmentation of motility and invasiveness* in vitro*, which can be transiently downregulated by treatment with antisense oligonucleotides to vimentin. Additional experimental evidence suggests that the mechanism(s) responsible for the differential expression of metastatic properties associated with the interconverted phenotype rest(s) in the unique interaction, either direct or indirect, of IFs with specific integrins interacting with the extracellular matrix [71].

The “motile phenotype” of cancer cells is expressed only through direct interactions with the tumor environment as inevitably the tumor cells will respond to local stimuli. These stimuli include cues for motility and migration, which normally appear in tissues undergoing formation, remodeling, or healing. Carcinoma cells are likely to be sensitive to the motility cues that normally regulate epithelial morphogenetic movements such as ingression, delamination, invagination, and tube or sheet migration [72]. Understanding how such motility cues arise and act, in tumor tissue, may provide one of the key “answers” in cancer research.

## 4. The Role of Matrix Molecules in Breast Cancer Cell Epithelial-to-Mesenchymal Transition

The huge proliferative ability of tumor cells leads to genetic diversity which facilitates their responsiveness to microenvironmental factors resulting in an increased degree of phenotypic plasticity [73, 74]. Therefore, during primary growth, some tumor cells can acquire traits that endow them with a malignant phenotype that leads to increased tumor cell motility, invasiveness, and propensity to metastasize [75]. Importantly, during epithelial-to-mesenchymal transition (EMT), tumor cells acquire a phenotype that encompasses all these traits as EMT is characterized by a loss of cell polarity and adhesion and gain of motile characteristics. Thus, the EMT promotes the detachment of cells from the primary tumor, facilitating their migration and metastatic dissemination [76]. Moreover, a strong link between EMT and acquisition of a tumor-initiating phenotype is suggested [77]. Early studies suggested the involvement of EMT in aggressive breast cancer behaviour as cells exhibiting a mesenchymal-like phenotype (vimentin expression, lack of cell border associated uvomorulin) show dramatically increased motility, invasiveness, and metastatic potential in nude mice [78]. Moreover, using an intravital imaging approach, Giampieri et al. showed that single breast tumor motile cells that have an active TGF-*β*-Smad2/3 EMT promoting signaling were capable of hematogenous metastasis to distal organs, while those lacking this signaling pathway were prone to passive lymph metastasis [79]. However, EMT is not the “ultimate” event as it involves various morphological and functional alterations [80] and is not always correlated to a more aggressive phenotype [81]. In addition, an apparent contradiction to the association between EMT and metastasis comes from clinical observations that distant metastases derived from a variety of primary carcinomas resemble an epithelial phenotype.

Importantly the EMT as well as the mesenchymal to epithelial transition (MET) is partly regulated through the “crosstalk” between the tumor microenvironment and the cancer cells [82]. Growth factor stimulation appears to be a part of this “crosstalk” as epidermal growth factor (EGF) leads to epitheliomesenchymal transition-like changes in human breast cancer cells including upregulation of vimentin and downregulation of E-cadherin. EMT was associated with increased ability of these cells to adhere to ECM molecules as well as to migrate [78]. Furthermore, TGF-beta1-mediated breast cancer invasion is associated with EMT and matrix proteolysis [83]. Likewise, constitutively active type I insulin-like growth factor receptor causes transformation and xenograft growth of immortalized mammary epithelial cells and is accompanied by an epithelial-to-mesenchymal transition mediated by NF-kappaB and snail [84]. Interestingly, TGF*β*-dependent hyaluronan synthase expression (HAS2) expression, but not extracellular hyaluronan, has an important regulatory role in TGF*β*-induced EMT [85]. Furthermore, when breast cells were induced to exhibit EMT, there was a strong upregulation of HAS2 [86].

Indeed, the implication of matrix molecules contribution to EMT was evident even from early studies [87]. LOX is a secreted amine oxidase that catalyses collagen and elastin cross-linking in the extracellular matrix, previously shown to regulate breast cancer metastasis, and is correlated to EMT [88]. Enhanced tenascin-C expression and matrix deposition during Ras/TGF-beta-induced EMT of mammary tumor cells was reported [89]. Noteworthy, there seems to be a shift in proteoglycans expression as significant correlation was found between the loss of the HSPG, SDC1, and epithelial expression during EMT. This loss was correlated with increased SDC1 stromal expression and a high grade of malignancy (*P* = 0.011). Therefore, the authors concluded that the loss of SDC1 epithelial expression was of strong prognostic value in breast carcinomas [90]. Along the same lines, SDC1 coexpression with E-cadherin was found to be synchronously regulated during EMT in breast cancer [91].

Importantly, the mesenchymal to epithelial transition (MET) of metastatic breast cancer cells upon reaching distant metastatic sites appears also to be regulated by ECM molecules as a unique paracrine crosstalk between the microenvironment and the cancer cells has been identified [92]. Thus, versican stimulated MET of metastatic breast cancer cells by attenuating phospho-Smad2 levels, which resulted in elevated cell proliferation and accelerated metastases. Analysis of clinical specimens showed elevated versican expression within the metastatic lung of patients with breast cancer [92]. Thus, mechanisms regulating both the EMT and MET processes are dependent on PG/GAG participation highlighting their relevance in breast cancer progression.

## 5. The Roles of GAGs/PGs in Breast Cancer Cell Motility

Breast cancer is characterized by significant quantitative changes of extracellular network constituents. Previously, it has been well established that changes in unique ECM properties of tumor cells and their microenvironment may lead to changes in cell behavior during cancer progression [12, 93]. The PG component of the ECM has been shown to participate in and regulate key cellular events, acting either directly on cells or modulating growth factor activities [94].

Thus, HSPGs are involved in multiple cellular events and functions such as cell adhesion, ECM assembly, and growth factors storage [95]. Their HS chains have the ability to bind not only to numerous “heparin-” binding growth factors and morphogens, [31] but also to “heparin-” binding sites present in matrix ligands, including fibronectin, vitronectin, laminins, and the fibrillar collagens [31]. The SDCs are believed to have roles in cell adhesion and signaling possibly as coreceptors with integrins and cell-cell adhesion molecules [96].

Each of the four SDCs has been proposed to connect to the actin cytoskeleton, via their cytoplasmic domains [97, 98], for example, through ezrin in SDC2 and a-actinin in the case of SDC4. For SDC1, SDC2, and SDC4 at least, the external core protein can trigger integrin-mediated cell adhesion events, which may be direct or, in the case of SDC4, probably indirect [98].

Many studies indicate a strong correlation between the expression of specific HSPGs and the metastatic and invasive potential of breast cancer cells [26, 99, 100]. In fact, the expression of SDC4 and the overexpression of SDC2 are associated with the high invasive potential of MDA-MB-231 cell line [29]. Interestingly, estradiol (E2) as well as IGF and EGF signaling pathways have significant roles in regulating the expression of certain cell surface HSPGs, such as SDC2, SDC4, and GPC1, which are crucial for cell motility [101].

SDC1 participates in the generation of a proangiogenic microenvironment, supporting tumor growth and metastatic spread [11, 12, 102]. This HSPG, regulates downstream signaling pathways that are traditionally associated with the integrins [12, 103, 104], thus mediating cell migration by creating a dynamic linkage between the ECM and the cytoskeleton and by modulating Rho family members that control the activation of focal adhesion kinase (FAK). Indeed, it has been observed that in MDA-MB-231 human breast cancer cells SDC1 physically interacts with FAK [105]. Furthermore, SDC1 regulates the activation of *α* *ν* *β*3 and/or *α* *ν* *β*5 integrins. This activation stimulates adhesion, spreading, and migration of tumor cells, with clear consequences on tumor progression [106]. Beauvais and Rapraeger, [106] also demonstrated that SDC1 collaborates with *α* *ν* *β*3 integrin to initiate a positive adhesion signal, which is integrin ligand independent. The activation of *β*1 integrins is not required for SDC1 mediated cell spreading; consequently SDC1 is sufficient for adhesion. Actually, the inhibition of *β*1 integrins activity induces cells spreading presumably by attenuating the suppression of SDC1 binding perpetrated by integrins. Additionally, SDC1 participates in the IGF-I receptor (IGF-IR) signaling pathway on adhesion. Specifically, it colocalizes with the integrin and IGF-IR and regulates activation of *α* *ν* *β*3 and *α* *ν* *β*5 integrins by coupling these integrins to the IGF-IR in human mammary carcinoma and endothelial cells, resulting in the activation of an inside-out signaling pathway [107]. Therefore, SDC1 is an obligatory component in the formation of this adhesion complex [107]. Additionally, SDC1 expression coordinates *β*-integrin dependent and interleukin-6 (IL-6) dependent cell functions, such as cell adhesion, migration, and resistance to irradiation, in MDA-MB-231 breast cancer cells [108].

SDC2 has likewise been implicated in cell adhesion and signaling [109] as well as in the progression of cancer [96]. The expression of SDC2 in breast cancer cells is regulated by estradiol (E2) through the action of estrogen receptor alpha (ER*α*) [110]. The increased levels of SDC2 after E2 treatment may be connected with the ability of SDC2 to modulate the tumorigenic and invasive behavior of breast cancer cells [110].

SDC3 has not been widely studied with respect to either breast or ovarian carcinoma [111], but its aberrant upregulation in vasculature associated with ovarian carcinoma has been noted [112].

SDC4 is a focal adhesion component in a range of cell types, adherent to different matrix molecules, including fibronectin [113, 114] and mediating breast cancer cell adhesion and spreading [103, 106]. The attachment of SDC4 to fibronectin triggers intracellular signaling, including protein kinase C*α* and focal adhesion kinase activation, to promote focal adhesion formation [115, 116]. SDC4 null cells are deficient in phosphorylated FAK and show impaired cell migration [116, 117]. When overexpressed, SDC4 promotes excess focal adhesion formation resulting in reduced cell migration [118]. Huang et al. [119] reported that tenascin-C, an adhesion-modulatory ECM molecule [120], binds to fibronectin (specifically to the FNIII13 of the HepII site), thereby specifically blocking cell adhesion to fibronectin through SDC4. This binding inhibits the coreceptor function of SDC4 in integrin signaling [119]. Nevertheless, the role of SDC4 on tumor progression needs more investigation, as different facets of its actions remain unclear.

An important feature of the SDC molecule necessary for signaling appears to be its ectodomain [96, 121]. Indeed, depleting epithelia of cell surface SDC1 alters cell morphology and organization, the arrangement and expression of adhesion molecules, and anchorage-dependent growth controls [121]. Therefore, Kato et al. [121] suggested a regulatory role for SDC1 ectodomain in the control of epithelial cell morphology. Soluble murine SDC4 ectodomain competes with the endogenous SDC4 for a critical cell surface interaction required for signaling during cell spreading [122, 123]. The ability of SDC4 to interact with molecules at the cell surface via its core protein as well as its GAG chains may uniquely regulate the formation of cell surface signaling complexes following engagement of this PG with its extracellular ligands [122, 123]. Moreover, shedding and membrane-associated SDC1 play distinct roles in different stages of ER*α*a+ breast cancer cell progression. Proteolytic conversion of SDC1 from a membrane bound into a soluble molecule marks a switch from a proliferative to an invasive phenotype, with implications for breast cancer diagnostics and potential GAG-based therapies [124].

A number of mutations related to SDCs have been recorded in breast carcinomas [111]. The mutations may influence the sequence of amino acids of the core protein and the enzymes that are involved in GAG chains synthesis. Importantly, these mutations may affect the interactions between SDCs and growth factors resulting in altered behavior of cells [125] including cell motility.

The expression of the GPC1 gene in the MDA-MB-231 may be indicative of its higher metastatic potential [29]. The expression of GPC3 is silenced in human breast cancer, but ectopic expression of GPC3 revealed that this molecule can act as a negative regulator of breast cancer cell growth [37, 39]. GPC3 may inhibit IGF and Wnt signaling, which are critical for cell motility and tumor progression, indicating that GPC3 may act as a metastasis suppressor [126, 127]. Another member of GPC family, GPC6, seems to have a key role in promoting the invasive migration of MDA-MB-231 cells through the inhibition of canonical-*β*-catenin and Wnt signaling and upregulation of noncanonical Wnt5a signaling through the activation of JNK (c-Jun-N-terminal kinase) and p38 MAPK (mitogen-activated protein kinase) [128]. Evidence suggests that GPCs are important in growth factor and morphogens responses, whereas roles in cell adhesion seem to be the prerogative of SDCs [111].

An important member of hyalectans, versican, is able to interact with ECM components and to bind to the cell-surface proteins CD44, integrin *β*1, and epidermal growth factor receptor (EGFR) [129, 130] to regulate cell processes such as adhesion, proliferation, migration, and ECM assembly [40, 130]. The expression of versican in breast carcinomas has been correlated to invasiveness [131]. Moreover, versican G3 domain enhanced breast cancer cell growth, migration, and metastasis by upregulating the EGFR mediated signaling pathways that contribute to a more metastatic phenotype [48]. Also, versican enhances breast cancer cell metastasis in mouse breast cancer cell lines, not only through facilitating cell motility and invasion but also by inhibiting preosteoblast cell growth and differentiation which supply favourable microenvironments for tumor metastases [132]. Enhanced understanding of the regulation and the involvement of versican in cancer may offer a novel approach to cancer therapy by targeting the tumor microenvironment [12].

The overexpression of decorin in the stroma of solid tumors counteracts cell growth, indicating that decorin may have a protective role in tumor progression [133]. Also, it seems to be a negative regulator for EGF signaling. Decorin's binding to EGFR initially leads to receptor's prolonged activation, followed by EGFR internalization and degradation, eliminating tumor growth and metastases [134]. Iozzo et al. [135] suggest that decorin loss may contribute to increased IGF-IR activity in the progression of breast cancer, where IGF plays a role on cell motility. Another member of SLRPs, lumican, may act as an inhibitor of migration, angiogenesis, and invasion by interfering with *α*2*β*1 integrin activity and downregulating MMP-14 expression to induce apoptosis [136]. Moreover, winter action of lumican with growth factors affects mobility, adhesion, and cell growth [137, 138].

Serglycin is the only characterized intracellular PG found in hepatopoietic and endothelial cells [12]. It carriers either heparan sulfate or chondroitin sulfate chains depending on cell type. Korpetinou et al. [139] have shown for the first time that serglycin is highly expressed in an aggressive breast cancer cell line (MDA-MB-231). The same authors demonstrated that the overexpression of serglycin promotes breast cancer cell growth, migration, and invasion [139]. Interestingly, overexpression of serglycin lacking the GAG attachment sites failed to promote these cellular functions, suggesting that glycanation of serglycin is necessary for its oncogenic properties. This study suggests that serglycin promotes a more aggressive cancer cell phenotype and may protect breast cancer cells from complement attack supporting their survival and expansion.

**Figure 1:**
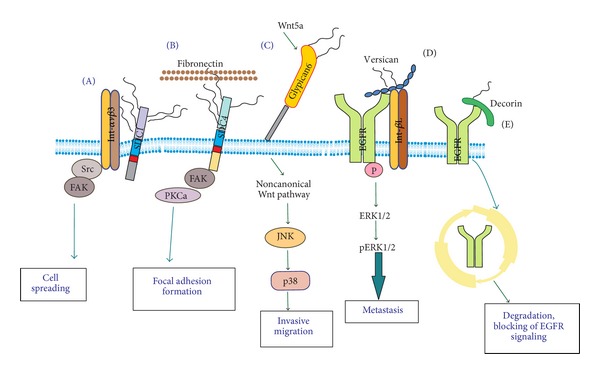
Roles of PGs/GAGs on the breast cancer motile phenotype. (A) Complex formation between syndecan1 (SDC1) and integrin-*α* *ν* *β*3 activate focal adhesion kinase to facilitate breast cancer cell spreading. (B) The specific binding of SDC4, through its HS chains, to fibronectin activates FAK/protein kinase Ca (PKCa) downstream signaling to initiate focal adhesion formation. (C) Wnt5a attaches to glypican6 in order to enhance breast cancer cell invasive migration through the involvement of noncanonical Wnt pathway and JNK/p38 downstream signaling. (D) Complex formation among versican, EGFR, and Int-*β*L activates extracellular matrix regulated kinase (ERK1/2) signaling to promote breast cancer metastasis. (E) Binding of decorin to EGFR causes receptor internalization, degradation, and subsequent inhibition of EGFR signaling.

HA is one of the principal ECM molecules and together with its CD44 cell surface receptor, it is implicated in cancer cell invasion and metastasis [62]. Indeed, high levels of HA are documented in malignant tumors, not only to the tumor stroma but also at the cell surface [62]. The elevated levels of the HA degrading, HYAL1 seems to regulate cell growth, adhesion, invasion, and angiogenesis of breast cancer [63]. Basal-like breast cancers (BL-BCa) have the worst prognosis of all subgroups of this disease. Indicatively, HA-induced CD44 signaling increases a diverse spectrum of protease activity including MTI-MMP and cathepsin K, to facilitate the invasion associated with BL-BCa cells, providing new insights into the molecular basis of CD44-promoted invasion [140]. Moreover, the cell surface HA, which is secreted by breast cancer cells, increases the adhesion ability of tumor cells, to lymphatic endothelial receptor (LYVE-1) [132]. Importantly, the molecular weight of HA seems to play a key role in the process of cell adhesion [141, 142], and particularly low molecular weight of HA promotes cell adhesion, while high molecular weight HA has no effect [143]. Indeed, LMW-HA plays an important role in CD44-TLR-associated AFAP-110-actin interaction and MyD88-NF-*κ*B signaling required for tumor cell behaviors, which may contribute to the progression of breast cancer [143]. The roles of PGs/GAGs on breast cancer cell motile phenotype are schematically depicted in Figure 1.

## 6. PGs/GAGs Potential Targets in Breast Cancer

In many* in vitro *studies breast cancer cells were treated with various anticancer agents, including inhibitors of tyrosine kinase receptors and other molecules, such as small peptides, which are related to the expression of proteoglycans, in order to observe changes in cell functions [29, 144, 145]. Thus, a new generation of bisphosphonate, zoledronate (zoledronic acid, Zometa), downregulates the expression levels of SDC1, SDC2, and GPC1 and upregulates the expression of SDC4 in breast cancer cells of low and high metastatic capability [144]. Furthermore, the downregulation in the expression of HA and its receptor CD44 which is directly associated with the migration and matrix-associated invasion of breast cancer cells was also observed [144]. Imatinib, a specific tyrosine kinase inhibitor, which targets PDGFRs, had a similar effect on breast cancer cells. Imatinib resulted in an inhibition of the PDGF-BB mediated expression of HSPGs, which is associated with its inhibitory effect on the invasive and migratory potential of breast cancer cells [29]. A different approach was utilized by Rapraeger [145]. These authors used a small peptide, synstatin, to target SDC1. Thus, the site in the SDC1 ectodomain that is responsible for capture and activation of the *α* *ν* *β*3 or *α* *ν* *β*5 integrins by IGF1R can be mimicked by this short peptide which competitively displaces the integrin and IGF1R kinase from the syndecan and inactivates the complex. The blocking in the formation of the receptor complex attenuates breast cancer cell metastasis [145].

It has been demonstrated that degradation of HS chains by heparanase 1 (HPSE-1) reveals cryptic HS fragments that play a significant role in controlling tumor cell growth and metastasis. It is thus likely that enzymatic degradation of HS could be used as a potential treatment against carcinogenesis since HS chains are involved in fundamental biological processes of both normal and metastatic cells [146]. Synthetic proteoglycans such as neoheparin and neoCS produced by carbodiimide (EDAC) conjugation of glycosaminoglycan (GAG) chains to a protein scaffold reduce cell viability by induction of apoptosis of myeloma and breast cancer cells* in vitro.* These results demonstrate the anticancer activities of this new class of GAG-based molecules [147].

In summary, this review focused on the roles of PGs/GAGs on breast cancer motility in order to identify possible therapeutic targets. The emerging mechanisms of PG/Gas action could potentially be exploited for designating discrete therapy targets for specific breast cancer grades.

## Abbreviations

(PG): Proteoglycan
(GAG): Glycosaminoglycan
(ECM): Extracellular matrix
(SLRPs): Small leucine-rich PGs
(GPC): Glypican
(SDC): Syndecan
(HYAL): Hyaluronidase
(HAS): Hyaluronan synthase
(EMT): Epithelial-to-mesenchymal transition
(MET): Mesenchymal to epithelial transition
(E2): Estradiol
(ERs): Estrogen receptors.

## References

[1] Nikitovic D, Papoutsidakis A, Karamanos NK, Tzanakakis GN (2014). Lumican affects tumor cell functions, tumor-ECM interactions, angiogenesis and inflammatory response. *Matrix Biology*.

[2] Nikitovic D, Kouvidi K, Karamanos NK, Tzanakakis GN (2013). The roles of hyaluronan/RHAMM/CD44 and their respective interactions along the insidious pathways of fibrosarcoma progression. *BioMed Research International*.

[3] Nikitovic D, Mytilinaiou M, Berdiaki A, Karamanos NK, Tzanakakis GN (2014). Heparan sulfate proteoglycans and heparin regulate melanoma cell functions. *Biochimica et Biophysica Acta*.

[4] Kouvidi K, Berdiaki A, Nikitovic D (2011). Role of Receptor for Hyaluronic Acid-mediated Motility (RHAMM) in Low Molecular Weight Hyaluronan (LMWHA)-mediated fibrosarcoma cell adhesion. *Journal of Biological Chemistry*.

[5] Mytilinaiou M, Bano A, Nikitovic D (2013). Syndecan-2 is a key regulator of transforming growth factor beta 2/smad2-mediated adhesion in fibrosarcoma cells. *IUBMB Life*.

[6] Chalkiadaki G, Nikitovic D, Katonis P (2011). Low molecular weight heparin inhibits melanoma cell adhesion and migration through a PKCa/JNK signaling pathway inducing actin cytoskeleton changes. *Cancer Letters*.

[7] Antonio JDS, Iozzo RV (2001). Glycosaminoglycans: Structure and Biological Functions. *eLS*.

[8] Syrokou A, Tzanakakis G, Tsegenidis T, Hjerpe A, Karamanos NK (1999). Effects of glycosaminoglycans on proliferation of epithelial and fibroblast human malignant mesothelioma cells: a structure-function relationship. *Cell Proliferation*.

[9] Mitropoulou TN, Tzanakakis GN, Nikitovic D, Tsatsakis A, Karamanos NK (2002). In vitro effects of genistein on the synthesis and distribution of glycosaminoglycans / proteoglycans by estrogen receptor-positive and -negative human breast cancer epithelial cells. *Anticancer Research*.

[10] Mendes de Aguiar CBN, Lobão-Soares B, Alvarez-Silva M, Gonçalves Trentin A (2005). Glycosaminoglycans modulate C6 glioma cell adhesion to extracellular matrix components and alter cell proliferation and cell migration. *BMC Cell Biology*.

[11] Afratis N, Gialeli C, Nikitovic D (2012). Glycosaminoglycans: key players in cancer cell biology and treatment. *The FEBS Journal*.

[12] Theocharis AD, Skandalis SS, Tzanakakis GN, Karamanos NK (2010). Proteoglycans in health and disease: novel roles for proteoglycans in malignancy and their pharmacological targeting. *FEBS Journal*.

[13] Iozzo RV (1998). Matrix proteoglycans: from molecular design to cellular function. *Annual Review of Biochemistry*.

[14] Schaefer L, Schaefer RM (2010). Proteoglycans: from structural compounds to signaling molecules. *Cell and Tissue Research*.

[15] Provenzano PP, Eliceiri KW, Campbell JM, Inman DR, White JG, Keely PJ (2006). Collagen reorganization at the tumor-stromal interface facilitates local invasion. *BMC Medicine*.

[16] Levental KR, Yu H, Kass L (2009). Matrix crosslinking forces tumor progression by enhancing integrin signaling. *Cell*.

[17] Lu P, Weaver VM, Werb Z (2012). The extracellular matrix: a dynamic niche in cancer progression. *Journal of Cell Biology*.

[18] Iozzo RV (1999). The biology of the small leucine-rich proteoglycans. Functional network of interactive proteins. *Journal of Biological Chemistry*.

[19] Andres JL, DeFalcis D, Noda M, Massague J (1992). Binding of two growth factor families to separate domains of the proteoglycan betaglycan. *Journal of Biological Chemistry*.

[20] Bernfield M, Kokenyesi R, Kato M (1992). Biology of the syndecans: a family of transmembrane heparan sulfate proteoglycans. *Annual Review of Cell Biology*.

[21] Leivonen M, Lundin J, Nordling S, von Boguslawski K, Haglund C (2004). Prognostic value of syndecan-1 expression in breast cancer. *Oncology*.

[22] Stanley MJ, Stanley MW, Sanderson RD, Zera R (1999). Syndecan-1 expression is induced in the stroma of infiltrating breast carcinoma. *American Journal of Clinical Pathology*.

[23] Maeda T, Alexander CM, Friedl A (2004). Induction of syndecan-1 expression in stromal fibroblasts promotes proliferation of human breast cancer cells. *Cancer Research*.

[24] Dhodapkar MV, Kelly T, Theus A, Athota AB, Barlogie B, Sanderson RD (1997). Elevated levels of shed syndecan-1 correlate with tumour mass and decreased matrix metalloproteinase-9 activity in the serum of patients with multiple myeloma. *British Journal of Haematology*.

[25] Yang Y, Yaccoby S, Liu W (2002). Soluble syndecan-1 promotes growth of myeloma tumors in vivo. *Blood*.

[26] Lendorf ME, Manon-Jensen T, Kronqvist P, Multhaupt HAB, Couchman JR (2011). Syndecan-1 and syndecan-4 are independent indicators in breast carcinoma. *Journal of Histochemistry and Cytochemistry*.

[27] Baba F, Swartz K, van Buren R (2006). Syndecan-1 and syndecan-4 are overexpressed in an estrogen receptor-negative, highly proliferative breast carcinoma subtype. *Breast Cancer Research and Treatment*.

[28] Malavaki CJ, Roussidis AE, Gialeli C (2013). Imatinib as a key inhibitor of the platelet-derived growth factor receptor mediated expression of cell surface heparan sulfate proteoglycans and functional properties of breast cancer cells. *FEBS Journal*.

[29] Mundhenke C, Meyer K, Drew S, Friedl A (2002). Heparan sulfate proteoglycans as regulators of fibroblast growth factor-2 receptor binding in breast carcinomas. *American Journal of Pathology*.

[30] Barbareschi M, Maisonneuve P, Aldovini D (2003). High syndecan-1 expression in breast carcinoma is related to an aggressive phenotype and to poorer prognosis. *Cancer*.

[31] Bernfield M, Götte M, Park PW (1999). Functions of cell surface heparan sulfate proteoglycans. *Annual Review of Biochemistry*.

[32] Lundström E, Sahlin L, Skoog L (2006). Expression of Syndecan-1 in histologically normal breast tissue from postmenopausal women with breast cancer according to mammographic density. *Climacteric*.

[33] Löfgren L, Sahlin L, Jiang S (2007). Expression of syndecan-1 in paired samples of normal and malignant breast tissue from postmenopausal women. *Anticancer Research*.

[34] Götte M, Kersting C, Ruggiero M (2006). Predictive value of syndecan-1 expression for the response to neoadjuvant chemotherapy of primary breast cancer. *Anticancer Research*.

[35] Tokes AM, Szasz AM, Farkas A (2009). Stromal matrix protein expression following preoperative systemic therapy in breast cancer. *Clinical Cancer Research*.

[36] Filmus J, Capurro M, Rast J (2008). Glypicans. *Genome Biology*.

[37] Xiang YY, Ladeda V, Filmus J (2001). Glypican-3 expression is silenced in human breast cancer. *Oncogene*.

[38] Gonzalez AD, Kaya M, Shi W (1998). OCI-5/GPC3, a glypican encoded by a gene that is mutated in the Simpson-Golabi-Behmel overgrowth syndrome, induces apoptosis in a cell line-specific manner. *Journal of Cell Biology*.

[39] Matsuda K, Maruyama H, Guo F (2001). Glypican-1 is overexpressed in human breast cancer and modulates the mitogenic effects of multiple heparin-binding growth factors in breast cancer cells. *Cancer Research*.

[40] Wight TN (2002). Versican: a versatile extracellular matrix proteoglycan in cell biology. *Current Opinion in Cell Biology*.

[41] Jeffs AR, Glover AC, Slobbe LJ (2009). A gene expression signature of invasive potential in metastatic melanoma cells. *PLoS ONE*.

[42] Nara Y, Kato Y, Torii Y (1997). Immunohistochemical localization of extracellular matrix components in human breast tumours with special reference to PG-M/versican. *Histochemical Journal*.

[43] Ricciardelli C, Brooks JH, Suwiwat S (2002). Regulation of stromal versican expression by breast cancer cells and importance to relapse-free survival in patients with node-negative primary breast cancer. *Clinical Cancer Research*.

[44] Ricciardelli C, Mayne K, Sykes PJ (1998). Elevated levels of versican but not decorin predict disease progression in early-stage prostate cancer. *Clinical Cancer Research*.

[45] Suwiwat S, Ricciardelli C, Tammi R (2004). Expression of extracellular matrix components versican, chondroitin sulfate,
tenascin, and hyaluronan, and their association with disease
outcome in node-negative breast cancer. *Clinical Cancer Research*.

[46] Canavese G, Candelaresi G, Castellano I, Mano MP (2011). Expression of proteoglycan versican in in situ breast lesions: relations between stromal changes, histotype, and invasion. *Pathology Research and Practice*.

[47] Yee AJM, Akens M, Yang BL, Finkelstein J, Zheng P, Deng Z (2007). The effect of versican G3 domain on local breast cancer invasiveness and bony metastasis. *Breast Cancer Research*.

[48] Du WW, Yang BB, Shatseva TA (2010). Versican G3 promotes mouse mammary tumor cell growth, migration, and metastasis by influencing EGF receptor signaling. *PLoS ONE*.

[49] Du WW, Yang BB, Yang BL (2011). Versican G3 domain modulates breast cancer cell apoptosis: a mechanism for breast cancer cell response to chemotherapy and egfr therapy. *PLoS ONE*.

[50] Kischel P, Waltregny D, Dumont B (2010). Versican overexpression in human breast cancer lesions: known and new isoforms for stromal tumor targeting. *International Journal of Cancer*.

[51] McEwan PA, Scott PG, Bishop PN, Bella J (2006). Structural correlations in the family of small leucine-rich repeat proteins and proteoglycans. *Journal of Structural Biology*.

[52] Schaefer L, Iozzo RV (2008). Biological functions of the small leucine-rich proteoglycans: from genetics to signal transduction. *Journal of Biological Chemistry*.

[53] Leygue E, Snell L, Dotzlaw H (2000). Lumican and decorin are differentially expressed in human breast carcinoma. *The Journal of Pathology*.

[54] Boström P, Sainio A, Kakko T, Savontaus M, Söderström M, Järveläinen H (2013). Localization of decorin gene expression in normal human breast tissue and in benign and malignant tumors of the human breast. *Histochemistry and Cell Biology*.

[55] Troup S, Njue C, Kliewer EV (2003). Reduced expression of the small leucine-rich proteoglycans, lumican, and decorin is associated with poor outcome in node-negative invasive breast cancer. *Clinical Cancer Research*.

[56] Reed CC, Waterhouse A, Kirby S (2005). Decorin prevents metastatic spreading of breast cancer. *Oncogene*.

[57] Santra M, Eichstetter I, Iozzo RV (2000). An anti-oncogenic role for decorin: down-regulation of ErbB2 leads to growth suppression and cytodifferentiation of mammary carcinoma cells. *The Journal of Biological Chemistry*.

[58] Leygue E, Snell L, Dotzlaw H (1998). Expression of lumican in human breast carcinoma. *Cancer Research*.

[59] Tammi RH, Kultti A, Kosma V, Pirinen R, Auvinen P, Tammi MI (2008). Hyaluronan in human tumors: pathobiological and prognostic messages from cell-associated and stromal hyaluronan. *Seminars in Cancer Biology*.

[60] Toole BP, Wight TN, Tammi MI (2002). Hyaluronan-cell interactions in cancer and vascular disease. *Journal of Biological Chemistry*.

[61] Jojovic M, Delpech B, Prehm P, Schumacher U (2002). Expression of hyaluronate and hyaluronate synthase in human primary tumours and their metastases in scid mice. *Cancer Letters*.

[62] Auvinen P, Tammi R, Parkkinen J (2000). Hyaluronan in peritumoral stroma and malignant cells associates with breast cancer spreading and predicts survival. *The American Journal of Pathology*.

[63] Tan JX, Wang XY, Li HY (2011). HYAL1 overexpression is correlated with the malignant behavior of human breast cancer. *International Journal of Cancer*.

[64] Wells A, Grahovac J, Wheeler S, Ma B, Lauffenburger D (2013). Targeting tumor cell motility as a strategy against invasion and metastasis. *Trends in Pharmacological Sciences*.

[65] Ridley AJ (2006). Rho GTPases and actin dynamics in membrane protrusions and vesicle trafficking. *Trends in Cell Biology*.

[66] Howe AK (2004). Regulation of actin-based cell migration by cAMP/PKA. *Biochimica et Biophysica Acta*.

[67] Gehler S, Ponik SM, Riching KM, Keely PJ (2013). Bi-Directional signaling: Extracellular matrix and integrin regulation of breast tumor progression. *Critical Reviews in Eukaryotic Gene Expression*.

[68] Zhao J, Guan J (2009). Signal transduction by focal adhesion kinase in cancer. *Cancer and Metastasis Reviews*.

[69] Hannigan G, Troussard AA, Dedhar S (2005). Integrin-linked kinase: a cancer therapeutic target unique among its ILK. *Nature Reviews Cancer*.

[70] Savagner P (2010). The epithelial-mesenchymal transition (EMT) phenomenon. *Annals of Oncology*.

[71] Hendrix MJC, Seftor EA, Chu Y, Trevor KT, Seftor REB (1996). Role of intermediate filaments in migration, invasion and metastasis. *Cancer and Metastasis Reviews*.

[72] Quaranta V (2002). Motility cues in the tumor microenvironment. *Differentiation*.

[73] Hanahan D, Weinberg RA (2000). The hallmarks of cancer. *Cell*.

[74] Marusyk AV, Almendro A, Polyak K (2013). Intra-tumour heterogeneity: a looking glass for cancer?. *Nature Reviews Cancer*.

[75] Marcucci F, Bellone M, Caserta CA, Corti A (2014). Pushing tumor cells towards a malignant phenotype: stimuli from the microenvironment, intercellular communications and alternative roads. *International Journal of Cancer*.

[76] Thiery JP, Acloque H, Huang RYJ, Nieto MA (2009). Epithelial-mesenchymal transitions in development and disease. *Cell*.

[77] Morel A, Lièvre M, Thomas C, Hinkal G, Ansieau S, Puisieux A (2008). Generation of breast cancer stem cells through epithelial-mesenchymal transition. *PLoS ONE*.

[78] Ackland ML, Newgreen DF, Fridman M (2003). Epidermal growth factor-induced epithelio-mesenchymal transition in human breast carcinoma cells. *Laboratory Investigation*.

[79] Giampieri S, Manning C, Hooper S, Jones L, Hill CS, Sahai E (2009). Localized and reversible TGF*β* signalling switches breast cancer cells from cohesive to single cell motility. *Nature Cell Biology*.

[80] Wicki A, Lehembre F, Wick N, Hantusch B, Kerjaschki D, Christofori G (2006). Tumor invasion in the absence of epithelial-mesenchymal transition: podoplanin-mediated remodeling of the actin cytoskeleton. *Cancer Cell*.

[81] Marsan M, van den Eynden G, Limame R (2014). A core invasiveness gene signature reflects epithelial-to-mesenchymal transition but not metastatic potential in breast cancer cell lines and tissue samples. *PLoS ONE*.

[82] Gao D, Vahdat LT, Wong S, Chang JC, Mittal V (2012). Microenvironmental regulation of epithelial-mesenchymal transitionsin cancer. *Cancer Research*.

[83] Zheng Q, Safina A, Bakin AV (2008). Role of high-molecular weight tropomyosins in TGF-*β*-mediated control of cell motility. *International Journal of Cancer*.

[84] Kim H, Litzenburger BC, Cui X (2007). Constitutively active type I insulin-like growth factor receptor causes transformation and xenograft growth of immortalized mammary epithelial cells and is accompanied by an epithelial-to-mesenchymal transition mediated by NF-*κ*B and snail. *Molecular and Cellular Biology*.

[85] Porsch H, Bernert B, Mehić M, Theocharis AD, Heldin C, Heldin P (2013). Efficient TGF*β*-induced epithelial-mesenchymal transition depends on hyaluronan synthase HAS2. *Oncogene*.

[86] Lien HC, Lee YH, Jeng YM, Lin CH, Lu YS, Yao YT (2014). Differential expression of hyaluronan synthase 2 in breast carcinoma and its biological significance. *Histopathology*.

[87] Kiemer AK, Takeuchi K, Quinlan MP (2001). Identification of genes involved in epithelial-mesenchymal transition and tumor progression. *Oncogene*.

[88] Thomas C, Karnoub AE (2013). Lysyl oxidase at the crossroads of mesenchymal stem cells and epithelial-mesenchymal transition. *Oncotarget*.

[89] Maschler S, Grunert S, Danielopol A, Beug H, Wirl G (2004). Enhanced tenascin-C expression and matrix deposition during Ras/TGF-*β*-induced progression of mammary tumor cells. *Oncogene*.

[90] Loussouarn D, Campion L, Sagan C (2008). Prognostic impact of syndecan-1 expression in invasive ductal breast carcinomas. *British Journal of Cancer*.

[91] Götte M, Kersting C, Radke I, Kiesel L, Wülfing P (2007). An expression signature of syndecan-1 (CD138), E-cadherin and c-met is associated with factors of angiogenesis and lymphangiogenesis in ductal breast carcinoma in situ. *Breast Cancer Research*.

[92] Gao D, Joshi N, Choi H (2012). Myeloid progenitor cells in the premetastatic lung promote metastases by inducing mesenchymal to epithelial transition. *Cancer Research*.

[93] Gialeli C, Theocharis AD, Karamanos NK (2011). Roles of matrix metalloproteinases in cancer progression and their pharmacological targeting. *The FEBS Journal*.

[94] Iozzo RV (1997). The family of the small leucine-rich proteoglycans: key regulators of matrix assembly and cellular growth. *Critical Reviews in Biochemistry and Molecular Biology*.

[95] Sarrazin S, Lamanna WC, Esko JD (2011). Heparan sulfate proteoglycans. *Cold Spring Harbor Perspectives in Biology*.

[96] Beauvais DM, Rapraeger AC (2004). Syndecans in tumor cell adhesion and signaling. *Reproductive Biology and Endocrinology*.

[97] Couchman JR (2003). Syndecans: proteoglycan regulators of cell-surface microdomains?. *Nature Reviews Molecular Cell Biology*.

[98] Couchman JR (2010). Transmembrane signaling proteoglycans. *Annual Review of Cell and Developmental Biology*.

[99] Manon-Jensen T, Itoh Y, Couchman JR (2010). Proteoglycans in health and disease: the multiple roles of syndecan shedding. *FEBS Journal*.

[100] Xian X, Gopal S, Couchman JR (2010). Syndecans as receptors and organizers of the extracellular matrix. *Cell and Tissue Research*.

[101] Tsonis AI, Afratis N, Gialeli C (2013). Evaluation of the coordinated actions of estrogen receptors with epidermal growth factor receptor and insulin-like growth factor receptor in the expression of cell surface heparan sulfate proteoglycans and cell motility in breast cancer cells. *FEBS Journal*.

[102] Yip GW, Smollich M, Götte M (2006). Therapeutic value of glycosaminoglycans in cancer. *Molecular Cancer Therapeutics*.

[103] Fears CY, Woods A (2006). The role of syndecans in disease and wound healing. *Matrix Biology*.

[104] Lambaerts K, Wilcox-Adelman SA, Zimmermann P (2009). The signaling mechanisms of syndecan heparan sulfate proteoglycans. *Current Opinion in Cell Biology*.

[105] Ibrahim SA, Yip GW, Stock C (2012). Targeting of syndecan-1 by microRNA miR-10b promotes breast cancer cell motility and invasiveness via a Rho-GTPase- and E-cadherin-dependent mechanism. *International Journal of Cancer*.

[106] Beauvais DM, Rapraeger AC (2003). Syndecan-1-mediated cell spreading requires signaling by *α*v*β*3 integrins in human breast carcinoma cells. *Experimental Cell Research*.

[107] Beauvais DM, Rapraeger AC (2010). Syndecan-1 couples the insulin-like growth factor-1 receptor to inside-out integrin activation. *Journal of Cell Science*.

[108] Hassan H, Greve B, Pavao MSG, Kiesel L, Ibrahim SA, Götte M (2013). Syndecan-1 modulates *β*-integrin-dependent and interleukin-6-dependent functions in breast cancer cell adhesion, migration, and resistance to irradiation. *FEBS Journal*.

[109] Tkachenko E, Rhodes JM, Simons M (2005). Syndecans: new kids on the signaling block. *Circulation Research*.

[110] Kousidou OC, Berdiaki A, Kletsas D (2008). Estradiol-estrogen receptor: a key interplay of the expression of syndecan-2 and metalloproteinase-9 in breast cancer cells. *Molecular Oncology*.

[111] Yoneda A, Lendorf ME, Couchman JR, Multhaupt HAB (2012). Breast and ovarian cancers: a survey and possible roles for the cell surface heparan sulfate proteoglycans. *Journal of Histochemistry and Cytochemistry*.

[112] Davies EJ, Blackhall FH, Shanks JH (2004). Distribution and clinical significance of heparan sulfate proteoglycans in ovarian cancer. *Clinical Cancer Research*.

[113] Woods A, Couchman JR (1994). Syndecan 4 heparan sulfate proteoglycan is a selectively enriched and widespread focal adhesion component. *Molecular Biology of the Cell*.

[114] Woods A, Longley RL, Tumova S, Couchman JR (2000). Syndecan-4 binding to the high affinity heparin-binding domain of fibronectin drives focal adhesion formation in fibroblasts. *Archives of Biochemistry and Biophysics*.

[115] Rapraeger AC (2000). Syndecan-regulated receptor signaling. *Journal of Cell Biology*.

[116] Woods A (2001). Syndecans: transmembrane modulators of adhesion and matrix assembly. *Journal of Clinical Investigation*.

[117] Cary LA, Chang JF, Guan J (1996). Stimulation of cell migration by overexpression of focal adhesion kinase and its association with Src and Fyn. *Journal of Cell Science*.

[118] Longley RL, Woods A, Fleetwood A, Cowling GJ, Gallagher JT, Couchman JR (1999). Control of morphology, cytoskeleton and migration by syndecan-4. *Journal of Cell Science*.

[119] Huang W, Chiquet-Ehrismann R, Moyano JV, Garcia-Pardo A, Orend G (2001). Interference of tenascin-C with syndecan-4 binding to fibronectin blocks cell adhesion and stimulates tumor cell proliferation. *Cancer Research*.

[120] G. Vollmer (1997). Expression of tenascin-C by human endometrial adenocarcinoma and stroma cells: heterogeneity of splice variants and induction by TGF-*β*. *Biochemistry and Cell Biology*.

[121] Kato M, Saunders S, Nguyen H, Bernfield M (1995). Loss of cell surface syndecan-1 causes epithelia to transform into anchorage-independent mesenchyme-like cells. *Molecular Biology of the Cell*.

[122] McFall AJ, Rapraeger AC (1997). Identification of an adhesion site within the syndecan-4 extracellular protein domain. *The Journal of Biological Chemistry*.

[123] McFall AJ, Rapraeger AC (1998). Characterization of the high affinity cell-binding domain in the cell surface proteoglycan syndecan-4. *Journal of Biological Chemistry*.

[124] Nikolova V, Koo C, Ibrahim SA (2009). Differential roles for membrane-bound and soluble syndecan-1 (CD138) in breast cancer progression. *Carcinogenesis*.

[125] Vlodavsky I, Elkin M, Ilan N (2011). Impact of heparanase and the tumor microenvironment on cancer metastasis and angiogenesis: basic aspects and clinical applications. *Rambam Maimonides Medical Journal*.

[126] Peters MG, Farías E, Colombo L, Filmus J, Puricelli L, Bal de Kier Joffé E (2003). Inhibition of invasion and metastasis by glypican-3 in a syngeneic breast cancer model. *Breast Cancer Research and Treatment*.

[127] Stigliano I, Puricelli L, Filmus J, Sogayar MC, de Kier Joffé EB, Peters MG (2009). Glypican-3 regulates migration, adhesion and actin cytoskeleton organization in mammary tumor cells through Wnt signaling modulation. *Breast Cancer Research and Treatment*.

[128] Yiu GK, Kaunisto A, Chin YR, Toker A (2011). NFAT promotes carcinoma invasive migration through glypican-6. *Biochemical Journal*.

[129] Wu YJ, La Pierre DP, Wu J, Yee AJ, Yang BB (2005). The interaction of versican with its binding partners. *Cell Research*.

[130] Theocharis AD (2008). Versican in health and disease. *Connective Tissue Research*.

[131] Skandalis SS, Labropoulou VT, Ravazoula P (2011). Versican but not decorin accumulation is related to malignancy in mammographically detected high density and malignant-appearing microcalcifications in non-palpable breast carcinomas. *BMC Cancer*.

[132] Du WW, Fang L, Yang W (2012). The role of versican G3 domain in regulating breast cancer cell motility including effects on osteoblast cell growth and differentiation in vitro—evaluation towards understanding breast cancer cell bone metastasis. *BMC Cancer*.

[133] Skandalis SS, Afratis N, Smirlaki G, Nikitovic D, Theocharis AD, Tzanakakis GN (2014). Cross-talk between estradiol receptor and EGFR/IGF-IR signaling pathways in estrogen-responsive breast cancers: focus on the role and impact of proteoglycans. *Matrix Biology*.

[134] Zhu J, Goldoni S, Bix G (2005). Decorin evokes protracted internalization and degradation of the epidermal growth factor receptor via caveolar endocytosis. *Journal of Biological Chemistry*.

[135] Iozzo RV, Buraschi S, Genua M (2011). Decorin antagonizes IGF receptor I (IGF-IR) function by interfering with IGF-IR activity and attenuating downstream signaling. *The Journal of Biological Chemistry*.

[136] Vuillermoz B, Khoruzhenko A, D'Onofrio M (2004). The small leucine-rich proteoglycan lumican inhibits melanoma progression. *Experimental Cell Research*.

[137] Brezillon S, Zeltz C, Schneider L (2009). Lumican inhibits B16F1 melanoma cell lung metastasis. *Journal of Physiology and Pharmacology*.

[138] Zeltz C, Brézillon S, Käpylä J (2010). Lumican inhibits cell migration through *α*2*Β*1 integrin. *Experimental Cell Research*.

[139] Korpetinou A, Skandalis SS, Moustakas A (2013). Serglycin is implicated in the promotion of aggressive phenotype of breast cancer cells. *PLoS ONE*.

[140] Montgomery N, Hill A, McFarlane S (2012). CD44 enhances invasion of basal-like breast cancer cells by upregulating serine protease and collagen-degrading enzymatic expression and activity. *Breast Cancer Research*.

[141] Dickinson LE, Ho CC, Wang GM, Stebe KJ, Gerecht S (2010). Functional surfaces for high-resolution analysis of cancer cell interactions on exogenous hyaluronic acid. *Biomaterials*.

[142] Yang C, Cao M, Liu H (2012). The high and low molecular weight forms of hyaluronan have distinct effects on CD44 clustering. *The Journal of Biological Chemistry*.

[143] Bourguignon LYW, Wong G, Earle CA, Xia W (2011). Interaction of low molecular weight hyaluronan with CD44 and toll-like receptors promotes the actin filament-associated protein 110-actin binding and MyD88-NF*κ*B signaling leading to proinflammatory cytokine/chemokine production and breast tumor invasion. *Cytoskeleton*.

[144] Dedes PG, Gialeli C, Tsonis AI (2012). Expression of matrix macromolecules and functional properties of breast cancer cells are modulated by the bisphosphonate zoledronic acid. *Biochimica et Biophysica Acta*.

[145] Rapraeger AC (2013). Synstatin: A selective inhibitor of the syndecan-1-coupled IGF1R-*α*v*β*3 integrin complex in tumorigenesis and angiogenesis. *The FEBS Journal*.

[146] Liu D, Shriver Z, Venkataraman G, El Shabrawi Y, Sasisekharan R (2002). Tumor cell surface heparan sulfate as cryptic promoters or inhibitors of tumor growth and metastasis. *Proceedings of the National Academy of Sciences of the United States of America*.

[147] Pumphrey CY, Theus AM, Li S, Parrish RS, Sanderson RD (2002). Neoglycans, carbodiimide-modified glycosaminoglycans: a new class of anticancer agents that inhibit cancer cell proliferation and induce apoptosis. *Cancer Research*.

